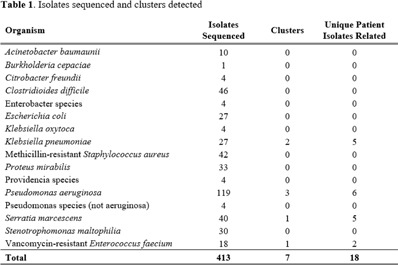# Monitoring disparities in healthcare-associated infection surveillance: A SHEA Research Network (SRN) Survey

**DOI:** 10.1017/ash.2022.212

**Published:** 2022-05-16

**Authors:** Caitlin McGrath, Valerie Deloney, Latania Logan, Lorry Rubin, Karen Ravin, Martha Muller, Allison Bartlett, Annabelle De St Maurice, Matthew Linam, Carolyn Caughell, Lynn Ramirez

## Abstract

**Background:** Inequities are seen in many health-related outcomes, and systemic and structural factors result in inequitable care based on social determinants of health (SDOH). However, whether disparities exist specifically in healthcare-associated infections (HAIs) based on these factors has not been well described. Furthermore, there are no national standards on whether information related to equity and SDOH should be included in HAI surveillance and how such information could be used. **Methods:** We surveyed US members of the SHEA Research Network (SRN), a consortium of healthcare facilities with leaders interested in healthcare epidemiology and infection prevention, via an online REDCap survey from October to December 2021. **Results:** Of the 68 eligible US SRN facilities, 28 (41%) responded. Among them, 27 institutions provide direct patient care and were analyzed. Of these 27 facilities, 8 (30%) collected data regarding variables related to equity including language for care, race or ethnicity, insurance status, and other. Of these faclilities, 38% are collecting but not otherwise using this information; other facilities use this information for a variety of reporting and intervention purposes (Fig. 2). Only 3 facilities (11%) analyzed whether disparities exist in any HAI rates. The most common barrier to collecting SDOH information is that facilities have not considered doing this work (Fig. 3). Of the 15 facilities not yet undertaking such work, 10 (67%) were interested in doing so. Specific recommendations about how to operationalize such collection are needed (Table 1). **Conclusions:** Most institutions in this sample are not collecting data that would allow for assessment of disparities in the rates of HAIs; however, there is interest in doing so. A minority of early adopter facilities are assessing whether disparities exist and are designing interventions. National guidance can play a key role in standardizing the collection of this information and translating early findings to identify and subsequently improve disparities within HAIs.

**Funding:** None

**Disclosures:** None

**Figure:**
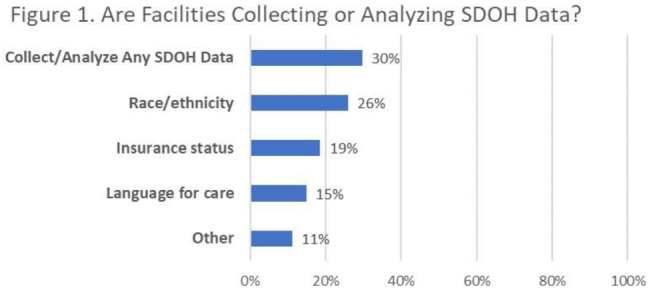

**Figure:**
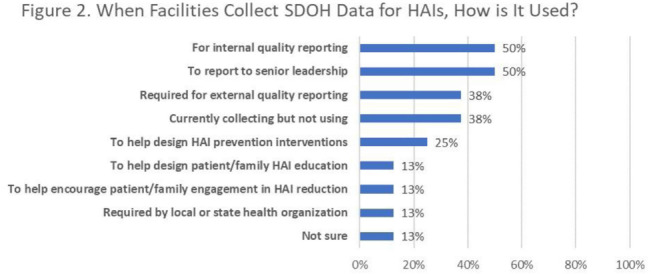

**Figure:**
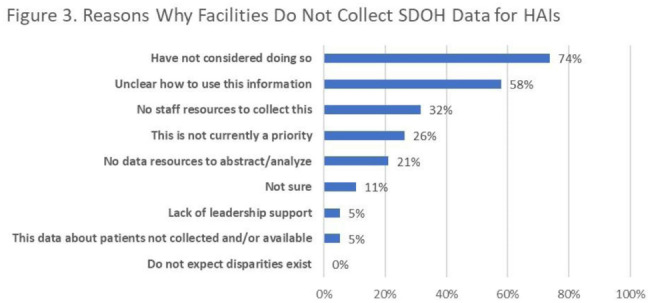

**Table:**